# Making sense of “senseless actions” in relation to criminal insanity

**DOI:** 10.3389/fpsyt.2026.1833835

**Published:** 2026-05-25

**Authors:** Søren Esben Rytter Heilskov, Julie Nordgaard, Unn Kristin Haukvik, Christine Friestad

**Affiliations:** 1 Centre for Research and Education in Forensic Psychiatry, Department of Mental Health and Addiction, Oslo University Hospital, Oslo, Norway; 2 Institute of Clinical Medicine, University of Oslo, Oslo, Norway; 3 Psychiatry East, Central and West Zealand Hospital, Roskilde, Denmark; 4 Department of Clinical Medicine, University of Copenhagen, Copenhagen, Denmark; 5 University College of Norwegian Correctional Service, Lillestrøm, Norway

**Keywords:** forensic psychiatry, irrationality, phenomenology, psychosis, schizophrenia, violence

## Abstract

In the assessment of criminal insanity, delusions play a particularly important role, because they express reality judgments, which, due to their verbalized form, serve well as “evidence” of psychosis. However, psychosis, understood as a fundamentally impaired sense of reality, can also involve disturbances that are neither verbalized nor inferential, but mainly *enacted*. In this paper, we revisit the psychopathological concept of “senseless actions”, employed by the German psychiatrist Klaus Conrad to describe the unintelligible actions observed in individuals in the early phase of schizophrenia. The forensic relevance of this concept is illustrated with a historical case study from the annals of Karl Wilmanns. We discuss how “senseless actions” express a global disturbance in the individual’s way of finding relevance, meaning, and constraint in the world, and argue that “senseless actions” may function as a behavioral indicator of the transition between a prodromal phase and manifest psychosis – pointing again to its forensic relevance. We stress the importance of considering contextual information and adopt a *Gestalt* view of how the actions inscribe themselves in the biography of the individual when determining whether “senseless actions” are indicative of psychosis. In conclusion, we suggest that the concept of “senseless action”, though imperfect and context sensitive, may offer a framework for identifying disturbances in reality understanding that risk being marginalized in contemporary approaches to criminal insanity.

## Introduction

Criminal insanity is a hybrid concept, composed of theoretical constructs from psychiatry, philosophy and science of law (1). The psychiatric literature’s most important theoretical contribution in this context is arguably the concept of psychosis. Thus, a criminal insanity evaluation usually revolves around the task of determining whether the individual was psychotic when committing the crime.

However, psychosis is itself a hybrid construct (2): If defined as a *fundamentally impaired sense of reality*, it follows that neither part of the definition can be understood, let alone operationalized, within a strict positivist bio-medical model, because the reality it refers to is not a reality ruled by the laws of *thermodynamics*, but by the dynamics of *social life*. In other words: while psychosis is conceptually rooted in a medical model of disease, its concept of reality is borrowed from the human sciences. In contemporary textbooks and diagnostic manuals, though, the difficult task of defining what psychosis *is*, is often solved by a “short circuit”-solution, defining psychosis as the presence of psychotic symptoms.

Among the psychotic symptoms, *delusions* play a particularly important role in the assessment of criminal insanity (3). One reason for this is that delusions, as expressions of irrationality, represent what Parnas (4) has termed “propositional irrationality”; a form of irrationality that reveals itself through explicit judgements about reality (propositions), e.g.: “my neighbor has secretly replaced my third molar with a radio device that broadcasts my thoughts”. Such statements may be subjected to a publicly shared analysis, e.g., of their internal logic, the plausibility of their content, the relation to the individual’s broader worldview and social milieu, and the way the individual draws inferences. Based on this analysis, one can argue whether the statement represents a degree of irrationality that warrants the label of psychosis.

Focusing on “propositional irrationality” does not eliminate ambiguity and interobserver disagreement – as was amply illustrated during the trial following the Norwegian 2011 mass murder committed by Anders Behring Breivik (5, 6). Nevertheless, the advantage of such statements is that by virtue of their verbalized form, they are easily made publicly available for scrutiny and discussion and can serve as tangible “evidence” of psychosis in court. For this reason, other psychotic symptoms (e.g.: hallucinations or catatonia) typically acquire their forensic significance only when “translated” into delusional beliefs, e.g., when an individual acts on instructions from voices which one believes come from God, etc.

However, a fundamentally impaired sense of reality does not necessarily involve harboring explicit false beliefs about external reality. In some instances, it may instead manifest in the form of what may be termed “non-propositional irrationality”. Mood disorders represent an uncontroversial example, in which a patient’s entire field of awareness is colored by the depressed/elevated mood. In this state, the patient’s *sense* of himself and his relation to the surrounding world may be fundamentally impaired by pervasive *feelings* of insufficiency and shame or conversely omnipotence and grandiosity. Another example could be severe formal thought disorders, which imply a breakdown of the shared reality-constituting platform of language. Such disturbances express a form of irrationality that reaches the threshold of psychosis, without in itself containing any explicit judgement (proposition) about reality.

These distinctions point to a structural asymmetry in psychiatric and forensic assessments: while propositional irrationality (e.g., delusions) can be articulated, scrutinized, and publicly evaluated through language, non-propositional irrationality resists such translation. As a result, forms of psychotic disturbance that do not present themselves as explicit beliefs risk being conceptually marginalized, despite potentially reflecting a profound disturbance in the individual’s grasp of reality.

When psychosis manifests as “non-propositional irrationality”, the disturbance is neither primarily verbal nor inferential, but enacted. Certain actions appear radically unintelligible in relation to the shared social world, yet cannot be adequately explained as expressions of delusional belief or goal-directed rationality. In the psychopathological literature, such phenomena have been described as “senseless actions” (7).

In a forensic context, one is occasionally confronted with instances of criminal behavior that in effect of their “senselessness” raise the suspicion of severe mental disorder. When no delusional motivation can be identified, however, such cases can easily fall in the conceptual gap between, on one side the colloquial reaction that “*this can’t be normal…*” (8) and on the other side a procrustean adherence to the so-called “operational” criteria of psychosis (6).

In this paper we revisit the psychopathological concept of “senseless action”, understood as the enactment of non-propositional irrationality, to examine its relevance for contemporary forensic psychiatric assessments of criminal insanity. We suggest that the concept of “senseless action”, despite its limitations, may represent a first step in bridging the conceptual gap by offering a theoretical framework that can be used to identify forms of insanity that are enacted rather than declared.

## The concept of “senseless action”

Strange, unreasonable or “senseless” behavior might arguably be considered the hallmark of insanity in a layman’s perspective. Yet as a phenomenon in its own right, it has received relatively little attention in psychopathological research and theory, which tend to focus instead on the suspected underlying causes of the behavior, e.g. delusions, hallucinations, catatonia.

The observation that psychosis may initially manifest through unintelligible actions can be found with Karl Jaspers (1883-1969):

“At the *start of a psychosis* behaviour often shows restlessness, haste, irresponsibility. There is an apparent lack of feeling for everything, which is suddenly interrupted by an outbreak of strong feeling; [ … ] there are sudden, unexpected actions, journeys, long walks in the night. It is as if the adolescent years have returned” (9).

Jaspers’ examples include wandering, self-mutilation – and homicide:

“An especially alarming event is the *meaningless murder* committed in the *preceding or initial stages of schizophrenia*. Motivation appears lacking, the deed is carried out with an unfeeling callousness, there is no insight or regret. The person talks with an alien indifference of what he has done. These really sick people have not been recognised as such by those around them and often not by their own doctor. They consider themselves quite well, but it is impossible really to understand what they have done. Only later on does diagnosis become certain” (9).

While Jaspers emphasized the “*un-understandable*” aspect of such actions, the Polish-French psychiatrist Eugène Minkowski (1885-1972) tried to understand how these actions relate to the individual’s overall altered mode of experience in psychosis. For Minkowski, who famously described the core disturbance of schizophrenia as a “loss of vital contact with reality”, the “unreasonable” actions of early psychosis, which he termed “délire en acte” (10), are particularly important for understanding the lifeworld of individuals with schizophrenia. In his view, these unintelligible actions represent an enactment of the “loss of vital contact with reality”, understood as a disruption of pre-reflective attunement to the implicit rules and dynamisms of social life (11). Thus, the individuals “act in the world without a sense for natural, contextual constraints or worldly demands” (10). To Minkowski, this disturbance needs not manifest itself in violence or other forms of antisocial conduct, but might result in rather subtle behavioral peculiarities or even in *inactivity*:

“[ … ] Another patient, formerly an engineer, no longer speaks at all, does not move, and only responds very briefly to attempts at conversation. He explains that he finds it preferable not to act at all because all his previous attempts to act turned out to be futile.” (10)

However, it is with the German psychiatrist Klaus Conrad (1905-1961) who coined the term “senseless action” (“Unsinnige Handlung”) that we find the most comprehensive account of the phenomenon (7). With few exceptions (12–14), Klaus Conrad’s work has received little attention in anglophone psychiatric research, and only fragments of his magnum opus – *Die beginnende Schizophrenie* (“The beginning schizophrenia”) has been translated into English (15). First published in 1958, the book is a synthesis of 107 cases of first-episode psychosis admitted to a military field hospital during the early years of World War II, from whom Conrad obtained painstakingly detailed self-description of the process of falling ill with psychosis. This phenomenologically inspired work resulted in what Conrad calls a “Gestalt-analysis” of the psychotic process. Gestalt-analysis in this context refers to an attempt to identify the defining changes in the *structure of experience* that emerges as a whole (*gestalt*) from the multitude of diverse individual cases.

According to Conrad, the phenomenon of “senseless action” can be observed in the early phase of psychosis, typically before the manifestation of other overt psychotic symptoms. Examples of “senseless actions” are sudden, aimless journeys, poorly motivated suicide attempts, and unruly behavior by previously exemplary soldiers. Taken together, actions that grossly violate contextual norms and are incomprehensible considering the individual’s previous conduct, to an extent that makes them appear “senseless” in the eyes of his peers. The use of the coarse and non-technical term “senseless” is deliberate, as is captures the quality of the actions; they appear to express a form of *radical irrationality* in a pre-theoretical, layman’s sense.

Jaspers’ designation of certain acts as “incomprehensible” concerns their resistance to reconstruction in terms of meaningful psychological motivation. Without disputing this limit, Conrad redirects the phenomenological analysis to a different level. Drawing on a Gestalt-analytic approach, he does not focus on the intelligibility of the action as such but examines how such acts emerge from a global alteration of the subject’s mode of experiencing self and world. In this way, actions that remain unintelligible at the level of motivation become phenomenologically intelligible as expressions of a transformed experiential Gestalt, without being reduced to motives or reasons.

According to Conrad, the prodromal phase of schizophrenia – which he refers to as the “trema” (an expression denoting the state of disquiet and nervous anticipation of the actor before entering the stage) – is characterized by a marked increase in “basic affectivity”, often articulated by the patient as a feeling of “tension” or “pressure”. In this phase, the patient has not yet formed any delusional *inferences* (cf. propositional irrationality) but does however *feel* that something in him or the surrounding world has changed – he *senses* that something is not right, that “there’s something in the air” (*Wahnstimmung*, “delusional mood”). An abyss opens between the patient and the world, which appears to him in a new, uncanny light. Meanwhile, a loosening or deformation of the individual’s frame of interpretation sets in: Seemingly neutral events or utterings are perceived by the individual with a special – often self-referring – significance.

In Conrad’s Gestalt-analysis, “senseless actions” are intimately related to these two phenomena – the increased affectivity and the altered perception of meaning – which he regards as aspects of an overall changed “structure of experience”. Thus, Conrad considers “senseless actions” as the individual’s attempt to relieve the tension through a “complete destruction of the situational structure”. The following is an example from Conrad of how a “senseless action” might manifest itself as self-directed violence:

Since June 1940, Case 91 had been anxious and agitated “like I never was before”. It was as if something was about to happen, as if a punishment was awaiting him, but he didn’t know what for. In January 1941, things got much worse; no one told him what was actually against him. But it was clear that something was against him. Ultimately, he was sick of being kept in the dark about everything. Then he did something stupid (namely, stabbing himself in the chest with a pocketknife). He had a strange feeling in his chest, as if it was too tight, as if his heart wasn’t with him. (7, our translation)

In this vignette, we see both the build-up of affective tension, the delusional mood (not yet articulated in organized delusional beliefs) characterized by an altered awareness of meaning, and finally the (self-directed) violent act as an idiosyncratic solution. In the *syncretism* (a concrete somatic sensation—tightness of the chest—is immediately fused with an abstract, existential meaning—”my heart wasn’t with me”—without clear differentiation between bodily and symbolic levels) and odd logic of the latter statement, we sense that it is not a question of acting according to empirically false beliefs about reality (propositional irrationality). Rather, it involves a global alteration in the individual’s way of finding meaning and in his overall “attunement” with reality (16).

Importantly, although “senseless actions” are often executed impulsively, they should not be reduced to poor impulse control or disinhibition. The apparent impulsiveness concerns the immediacy of the act, whereas the act itself is embedded in—and expressive of—the more pervasive transformation of the subject’s experiential *Gestalt*. In Conrad’s account, the impulsive quality of such actions thus reflects the alteration of the experiential field in the prodromal state, rather than a breakdown of volitional control.

## Karl Wilmanns’ case of Robert Engelbrecht

We now turn to yet another mid-20^th^ century German text to illustrate how phenomena resembling “senseless action” may manifest in the form of interpersonal (homicidal) violence. Karl Wilmanns (1873-1945) is primarily remembered for his work as professor of psychiatry in Heidelberg from 1918 to 1933, where he successfully gathered some of the most brilliant scholars of the time in the intersection between psychiatry and philosophy, such as Karl Jaspers and Hans W. Gruhle.

Although Wilmanns himself was a prolific writer, most of his work has not been translated into English, with one recent exception (17). In addition to his interest in social psychiatry and the study of mentally ill vagrants, Wilmanns was also engaged with questions in forensic psychiatry.

After being removed from his position at the Heidelberg clinic by the national socialist regime in 1933, Wilmanns published two essays, one of which is the 1940 text *Über Morde im Prodromalstadium der Schizophrenie* (“On Murders in the prodromal stage of schizophrenia”) (18). In this text, Wilmanns presents 26 cases of murders or attempted murders that he interpreted as having been committed by individuals in the early phase of psychosis. Some of the cases derive from Wilmanns’ own clinical experience, whereas others are based on historical cases, courts reports, and newspaper accounts.

The following excerpt is the first case of the essay, presented here in its entirety. The translation from German is our own.

On a Sunday in March 1920, at 12 noon, 23-year-old student Robert Engelbrecht shot 19-year-old student Kahn, whom he barely knew, with a Browning pistol in a crowded square in Baden-Baden. He then fired a second shot into his own thigh, sank to the ground and fired several more shots at the pavement. The perpetrator was immediately arrested and taken away. When his deeply shaken father visited him in the remand prison shortly afterwards and asked him in great consternation “Son, what have you done?”, Engelbrecht calmly replied, “*I wanted to become a murderer*.”

The perpetrator, who had a strong familial predisposition to schizophrenia on his mother’s side, was a gentle, lovable child with above-average intellectual abilities. In the upper classes of secondary school, however, his performance declined and he became inattentive, distracted and dreamy. During the World War, the slow transformation taking place within him became more pronounced. As evidenced by the letters send to his parents, he fluctuated greatly in his self-assessment; at times he considered himself a military genius, at other times he despaired of himself and often had the urge to take his own life. During a leave of absence in 1915, this previously war-enthusiastic, brave daredevil made a quiet, apathetic, depressed impression. He reported difficulty maintaining a coherent conversation and complained that his thinking was disturbed. His letters, which had previously been mostly short, factual and clear, took on a completely different character in the course of 1916; in them, he discussed his new, unclear, and contradictory worldview with a peculiar self-importance. At the same time, his mood swings became more pronounced. Periods of profound depression, during which he despaired of himself, withdrew and avoided all contact, alternated with episodes of cheerful spirit, in which he indulged in drinking, rebelled against his superiors and engaged in childish pranks.

By the end of the war, his condition deteriorated considerably. E. became increasingly quiet and withdrawn, isolated himself and ceased to socialize. He immersed himself in religious and political reading, and constantly changed his career plans, at various times studying medicine, history, economics, philosophy, veterinary medicine, and zoology, but never persisting in any of them. Ultimately, he abandoned the idea of professional training altogether.

His plans became increasingly fantastical. At one point, he wanted to renounce everything and live in poverty and hardship, earning his living as a simple farmer or woodcutter; at another, he planned to emigrate to Russia and join the Red Army to fight against England. At times, he was seized by the idea that he was called to higher things and should not shirk his mission from God; he planned to found a new religion in Russia as the Messiah, a “conservative Bolshevism”. When he discussed his political and religious enlightenment with his relatives, he displayed arrogance, volatility, and distraction, and became ecstatic; if he encountered opposition, he fell into a state of violent agitation, throwing plates against the wall and rolling on the floor in despair.

The act caused great public excitement, and various rumors circulated about his motive, including suggestions that he had acted out of anti-semitism, jealousy, and the like. All of these rumors proved to be false. The act remained a mystery. The suspicion that E. had acted in a state of mental disturbance was obvious and proved to be well-founded.

E. was – as already mentioned – originally a highly intelligent young man of impeccable character and unconditional sincerity and truthfulness. However, he felt no remorse, but thought a great deal about the act, which he himself did not understand. “*Since*, “ as he once said, “*there are no words to describe the tension of the emotional experience, “* his statements about his inner state before, during, and after the act should therefore be seen only as attempts to convey, to himself, the doctor, and the judge, the incomprehensible and indescribable state of emergency in which he found himself. The result of his efforts is all the more instructive because he was a psychologically perceptive person and genuinely sought to understand his own behavior, which remained alien and incomprehensible to him.

E. had for years been suffering from “*states of unbearable inner tension*”. Even during the war, he had occasionally felt an urge to take his own life, and recently he had often felt compelled to “*bring about some kind of reaction*”, to burn himself in a fire, to shoot out his eyes, to commit murder, in short, to commit some act “*from which there would be no turning back*”.

On that terrible day, he did not initially think of committing the act. Although he felt “*an indefinable agitation that he could not control*” throughout the morning, he hoped to find distraction and reassurance with a friend and left the house. However, when she did not receive him, his agitation increased. He wandered through the streets while contradictory thoughts and feelings arose and quickly faded again. When the strange urge to “*break free from the unbearable tension and anxiety in some way*” suddenly returned, the thought of killing someone came over him again.

He now decided to “*use the impulse to regain his inner freedom*” and hoped “*to find the strength for revelation in the agony and fear of murder*.” When he returned home to fetch his revolver, he did not attract the attention of any witnesses. On the contrary, he seemed cheerful and in good spirits. He then set off towards the city center. Along the way he hesitated to carry out his plan, and wavered for a long time about whom he should choose as his victim: an old man, a young husband, a policeman or someone else, until the student, whom he hardly knew, happened to cross his path.

Now he followed the impulse *“like a puppet*” and shot K. in broad daylight on a busy street. “*The idea of a prophetic role*” had been “*the conscious motive behind the whole action, “* and “*the idea of the Messiah*” was still alive in him immediately after the deed. Now he felt “*as if he were awakening to reality”* and realized that he had not achieved “*inner liberation.”* He immediately made a full confession before the judge, rejected a defense lawyer and demanded the punishment he deserved. E. was acquitted and transferred to a mental institution, where he soon fell into a stormy schizophrenia and his personality completely disintegrated. (18, our translation) (This extract is under copyright of Springer Nature, dec. 31, 1969. Published with permission.)

Wilmanns’ case of *Robert Engelbrecht* highlights many recognizable aspects from Conrad’s descriptions: The “unbearable inner tension” and “indefinable agitation” combined with the gradual change in the individual’s sense of his own significance in relation to the surrounding world. Through the patient’s descriptions, the homicidal act appears to resist interpretation both as meaningfully motivated (jealousy, political radicalism) and as emanating from elaborated delusional beliefs. Rather, the “radical irrationality” (19), so clearly expressed in the case, cannot be inferred from any single fact or statement, but transpires as a *Gestalt* arising from the matrix of his life history, his self-descriptions–and not least his actions.

## Discussion

In this paper, we have revisited the psychopathological concept of “senseless actions” and illustrated how such actions may manifest as violent behavior in the early phases of psychotic illness. Rather than approaching these acts as epiphenomena of delusional belief or failures of impulse control, we have argued that they can be understood as enactments of a disturbed reality-understanding that is not primarily propositional in nature. This distinction has important forensic implications. Because contemporary assessments of criminal insanity tend to privilege verbally expressible forms of irrationality, most notably delusional beliefs, forms of psychotic disturbance manifesting primarily through action risk being overlooked or misclassified.

Descriptively, “senseless actions” are actions that transgress contextual norms and resist empathic understanding within a given social setting. Crucially, this senselessness is not an intrinsic property of the action itself but arises from its relation to the individual’s prior life history, situational context, and mode of engagement with the shared social world. As Conrad’s military cases illustrate, the threshold for what appears “senseless” is context-sensitive: within a rigidly structured environment, even relatively minor deviations may acquire diagnostic salience (7). Allowing for this contextual variability, the defining feature of “senseless actions” lies not in norm violation as such, but in the way the actions express a more global disturbance in the individual’s way of finding relevance, meaning, and constraint in the world (19).

A striking feature emphasized by both Jaspers, Wilmanns, and Conrad is that “senseless actions” tend to occur early in the course of illness, often before the emergence of fully developed psychotic symptoms (7, 9). In their clinical examples, neither the individuals themselves nor their surroundings recognized the presence of a severe mental disorder prior to the act that retrospectively raised suspicion of psychosis. As has been noted in empirical research on prodromal and high-risk states, early symptoms are often heterogeneous, non-specific, and difficult to assess prospectively (20). In many cases, their significance becomes clear only retrospectively, once a full psychotic disorder has developed (21). This temporal ambiguity is particularly relevant in forensic contexts, where the interpretation of actions occurring at early stages of illness may depend on whether they are understood as part of an emerging psychotic process or as occurring outside of it. The concept of “senseless actions” could arguably have a twofold relevance in this context: First, it establishes a meaningful connection between the criminal actions and the experiential changes of the psychotic process. Secondly, it may function as behavioral indicator of a transitional phase, situated between prodromal disturbance and manifest psychosis. For forensic psychiatry—tasked with applying categorical distinctions such as psychotic versus non-psychotic to experiential processes that appear inherently fluid —this temporal positioning is of particular relevance.

While the relationship between delusions and actions is far from straightforward–often characterized by a peculiar “inconsequentiality” (22)–the prevailing view in clinical psychiatry tends to treat action as the ultimate “proof” of the reality status of psychotic experience. This view is reflected, for example, in the Positive and Negative Syndrome Scale (PANSS), where acting in accordance with the content of delusions or hallucinations is assigned the highest ratings (23). One could argue that a similar principle applies in cases of irrationality that resist verbalization in the form of propositional statements. Here, the actions themselves are “statements” expressing the individual’s understanding of reality.

It must be emphasized that “senseless actions” are not synonymous with psychosis, nor can they serve as direct evidence of criminal insanity. Disturbances of attunement to social reality may exist below the threshold of psychosis and can, as mentioned earlier, manifest in subtle behavioral peculiarities, withdrawal, or inactivity rather than violence (10, 24). Thus, the forensic relevance of “senseless actions” cannot be established by the action in isolation, but requires a Gestalt-assessment that situates the act within the individual’s life history, experiential changes, and overall mode of being-in-the-world (7).

This distinction also clarifies how senseless actions differ from other forms of maladaptive or violent behavior. Most criminal acts, particularly acts of interpersonal violence, conflict with contextual norms and may appear “irrational” in a colloquial sense. Yet actions driven by jealousy, rage, or spite typically remain intelligible within the shared horizon of human motivations, however morally troubling they may be. Such actions still presuppose continued participation in a common reality, structured by recognizable meanings and emotional logics, which also accommodates a considerable degree of “irrationality” (11). By contrast, “senseless actions” enact a more radical displacement from this shared space of meaning. Borrowing Conrad’s metaphor, the individual does not merely break the rules of the game; rather, the entire game board has been overturned (7).

Recognizing such disturbances places significant demands on forensic psychiatric practice. It requires not only a Gestalt-oriented clinical perspective, but also theoretical familiarity with forms of psychopathology that do not present themselves as explicit beliefs. Moreover, it requires a willingness to suspend the “principle of charity”, which denotes the tendency to ascribe rational motives to actions whenever possible (25). While such charity is indispensable in ordinary social life, applying it uncritically in forensic settings risks obscuring precisely those forms of irrationality that do not conform to familiar motivational patterns.

In a forensic context, however, identifying such disturbances in the individuals’ understanding of reality is only one side of the coin: In addition, the forensic expert should be able to communicate the assessment in a manner that allows the court to match this against the legal concept of criminal insanity, which is partially overlapping with (but not identical to) the psychiatric concept of psychosis. In practice, this often amounts to listing which symptoms are present, with delusions taking precedence. The resulting situation is somewhat paradoxical, since the kinds of disturbances in reality understanding that are perhaps the most obvious in a layman’s perspective (the case of Robert Engelbrecht being a splendid example), are in many jurisdictions not likely to weigh in in the evaluation of criminal insanity, because they cannot be translated into delusional thought content.

The challenge facing psychiatry, as a scientific and forensic discipline, is therefore not merely to operationalize ever more refined symptom categories, but to develop conceptual frameworks capable of addressing disturbances that resist straightforward verbalization. The concept of “senseless actions” represents one such framework, imperfect and context-sensitive, yet potentially indispensable when confronting forms of insanity that are enacted rather than declared.

An important task for future research, lies in exploring the boundaries between “senseless actions” and aberrant behavior observed in other conditions entailing disturbances in pre-reflective intersubjective attunement, e.g. autism spectrum-disorders (26).

In conclusion, we suggest that “senseless actions” can be understood as potential behavioral manifestations of disturbed intersubjective attunement, often associated with, but not reducible to, psychotic illness. The concept does not offer a shortcut to diagnosing psychosis or criminal insanity, nor does it replace established forensic criteria. Rather, its value lies in drawing attention to a dimension of psychopathology that is easily marginalized when assessments focus exclusively on forms of irrationality that can be translated into delusional thought content. Without such attention, forensic psychiatry risks presenting an incomplete picture of an individual’s understanding of reality at the time of the offense.
